# Fluticasone Propionate-loaded Black Cumin Oil NLCs with Dual Deposition/Permeation Capabilities for Enhanced Psoriasis Management

**DOI:** 10.34172/apb.47080

**Published:** 2026-05-19

**Authors:** Mohamed Ashraf, Hossam S. El-Sawy, Ghada M. El Zaafarany, Mona M. A. Abdel-Mottaleb

**Affiliations:** ^1^Department of Pharmaceutics and Pharmaceutical Technology, Faculty of Pharmacy, Egyptian Russian University, Cairo 11829, Egypt; ^2^Department of Pharmaceutics, College of Pharmacy, University of Kut, Wasit, 52001, Iraq; ^3^Department of Pharmaceutics and Industrial Pharmacy, Faculty of Pharmacy, Ain Shams University, Cairo 11566, Egypt; ^4^Université de Franche-Comté, EFS, INSERM, UMR 1098 RIGHT, Besançon, France

**Keywords:** Psoriasis, Black cumin oil, Nanostructured lipid carriers, Fluticasone propionate, Imiquimod model, Inflammatory cytokines

## Abstract

**Purpose:**

This study aimed to enhance the therapeutic effect of fluticasone (FP) for psoriasis using black cumin oil-based nanostructured lipid carriers (NLCs) with dual deposition/permeation capabilities to achieve high drug localization within the viable skin layers (deposition) for targeted topical action, while concurrently facilitating efficient drug flux through the skin (permeation) to address the full depth of psoriatic plaques.

**Methods:**

Oil selection followed a preliminary NLC optimization study. Characterization used microscopy, FTIR, DSC, ex vivo skin permeation and deposition, and in vivo pharmacodynamics with imiquimod-induced psoriatic mice.

**Results:**

The optimized NLC showed a size of 171.3 nm, a PDI of 0.37, and a zeta potential of -32.3 mV. Ex-vivo data showed dual local/systemic distribution, with FP deposition in the stratum corneum and epidermis, plus superior permeation versus marketed cream. An in vivo study revealed PASI score improvement up to 77%. IL-6 and IL-1α levels were significantly reduced. Histology showed inflammation decline.

**Conclusion:**

Results demonstrated improved anti-psoriatic action using the dual distribution/combinatorial approach with the optimized formulation. The data confirmed that the black cumin oil-based NLC formulation offers better outcomes than the standard cream by enhancing FP skin targeting, penetration, and anti-inflammatory response in psoriatic models.

## Introduction

 Psoriasis is a common, complex dermatological autoimmune disorder.^1^ It usually appears in the form of reddened, raised lesions with silvery or white scales. It is also characterized by abnormal epidermal structure of skin, differentiation of keratinocytes, and an increasing rate of keratinocyte turnover.^2^ The name “psoriasis” comes from the word *Psora, *a Greek-origin word that means (Itch).^3^ Psoriasis is mostly triggered by environmental or genetic factors.^4^ Psoriasis treatment can be classified into four broad categories: topical treatment including corticosteroids, vitamin D analogues, keratolytics, and retinoids; UV therapy. Topical therapy is considered first-line therapy for mild to moderate cases and can be used in combination with systemic drugs (methotrexate and cyclosporins) and biologics.^5^ While topical treatment offers benefits like patient compliance and potential efficacy, it also has limitations due to scaly, horny skin. A reduction in psoriasis area and severity index (PASI) score is a measure of the efficacy of treatment. Side effects of topical treatment were reported, such as skin atrophy and skin irritation, while systemic treatment can cause long-term suppression of the immune system and kidney dysfunction over time.^6^

 Nanotechnology-based drug delivery systems aim to improve efficacy and patient compliance and reduce side effects by increasing drug retention in targeted skin layers and controlling release.^7^ Lipid-based colloidal carriers are biodegradable, non-toxic lipids, forming lipid-vesicular or lipid particulate carriers. Liposomes, transferosomes, and niosomes are spherical shells, while nanostructured lipid carriers and solid lipid nanoparticles are lipid matrix-based carriers.^8^ Nanostructured lipid carriers (NLCs) contain both solid and liquid lipids, which allow more space for drug loading into their matrix and minimal expulsion of drugs. NLC, produced through high-shear homogenization and high-speed stirring, offers biocompatibility, close contact to stratum corneum, and enhanced drug penetration through lipid exchange and SC fluidization.^9^ In 2019, a study utilized a hot melt homogenization method to prepare dithranol-loaded NLC, demonstrating enhanced drug penetration, controlled release, plaque reduction, and reduced inflammatory mediators in psoriasis treatment.^10^ Another investigation aimed to study the permeation of the econazole NLC gel system. It showed higher permeability and enhanced flux rate, which resulted in an improved therapeutic outcome for skin fungal infections.^11^ NLC formulations containing Emulgin, orange wax, and rice bran wax were prepared for enhancing the skin penetration of lycopene. NLC participated in increasing the stability of lycopene and retarding its degradation.^12^ Microemulsion technique was used to design NLC with mometasone furoate for psoriasis treatment, enhancing drug permeation and skin deposition by 2.5 times compared to marketed formulations.^13^ Mentioned advantages of NLC such as biocompatibility, biodegradability, improved permeation and deposition through targeted skin layers, increasing contact with SC, and higher drug loading establish the NLC system as an optimum topical drug delivery system.

 Black cumin (*Nigella sativa)* oil, which has a wide spectrum of pharmacological activities, is the main component of an herbaceous plant belonging to the family Ranunculaceae. Ethanolic extract of black cumin seeds proved its efficacy in overcoming psoriasis comparable to standard positive control tazarotene.^14^ Black cumin essential oil, rich in thymoquinone, linoleic acid, oleic acid, and palmitic acid, has anti-inflammatory properties by reducing cytokine burden and inhibiting keratinocyte proliferation, potentially reducing psoriasis and inflammation.^15^ *Nigella sativa* ointment was proven to have an excellent anti-psoriatic curative effect with a low relapse rate alone or in combination with an oral dose with no observed side effects.^16^ It was also proved that thymoquinone (main component) of black cumin oil has immunomodulatory effects and anti-inflammatory properties.^17^ A study developed a nanoemulsion formulation using black cumin oil to deliver tacrolimus topically to treat psoriasis, showing minimal systemic exposure and significant healing effects.^18^ Eucalyptus and tea tree oil, containing cineole, linalool, and p-cymene, have been found to alleviate inflammatory conditions by reducing cytokine mediators and inhibiting inflammatory cytokine production.^19,20^

 Coconut oil, derived from the coconut kernel, regulates cytokine expression, alleviating inflammation symptoms and potentially managing psoriasis. Its solid nature allows it to be used in NLCs, with high solubility reaching 4.21 mg/ml. It also enhances skin barrier functions.^21^ β-sitosterol-loaded coconut oil NLCs were prepared by using a high-speed homogenization method. The oil phase was composed of glyceryl monostearate, coconut oil, and Tween 80 as a surfactant; the optimized formulation showed enhanced effective delivery of lipophilic β-sitosterol and improved entrapment efficiency, which was further packed into microneedles for treating androgenic alopecia.^22^

 Fluticasone propionate (FP) is a potent topical corticosteroid with high lipophilicity, receptor affinity, and binding/retention properties, effectively disrupting nuclear factor kappaB (NF κB) to stop inflammation. It inhibits the activation and proliferation of neutrophils and lymphocytes, cytokine generation, and TNF-α-induced adhesion molecule expression. In addition, FP does not inhibit the hypothalamus-pituitary-adrenal axis and is safe and well-tolerated in individuals of all ages.^23^ However, skin sensitivity and local skin adverse effects such as pustules, pruritus, burning, irritation, exacerbation of dermatological conditions, and folliculitis may appear with long-term use of FP.^24^ Another major obstacle in topical treatment using corticosteroids is the reduced efficacy with long-term use during psoriasis treatment, which is a resistant disease.^25^

 So, we aim in this work to formulate NLC using a mixture of solid lipid (coconut oil) and another liquid lipid with reported anti-inflammatory effects acting as pharmacologically synergistic excipients with FP corticosteroid to enhance its therapeutic effect against psoriasis. The prepared NLCs and the physicochemical interactions of the solid/liquid lipid components were evaluated for their influence on particle size, PDI, and zeta potential, as well as surface and morphological aspects of NLCs and ultimately impact on efficacy *in vivo.* The optimized selected formula’s morphological aspects were then evaluated using transmission electron microscope (TEM) imaging. They were also characterized using Differential Scanning Calorimetry (DSC) and Fourier Transform Infrared (FTIR) spectroscopy. Moreover, *ex vivo* skin permeation and deposition experiments were performed, and finally, the therapeutic efficacy of the system was evaluated *in vivo* using imiquimod-induced psoriasis in mice.

## Materials and Methods

###  Materials

 Fluticasone propionate USP was a kind gift from MUP Company (Cairo, Egypt). Tween 80 was purchased from Adwic-Al Nasr Pharmaceutical Chemical Company (Cairo, Egypt). Polyethylene glycol 400 was purchased from Piochem (Cairo, Egypt). Coconut oil was purchased from National Company (Cairo, Egypt). Eucalyptus oil was purchased from Nefertari company (Cairo, Egypt). Black cumin oil and tea tree oil were purchased from Health and Food Company (Cairo, Egypt). Acetonitrile and ethanol (HPLC grade) were purchased from Fisher Scientific (New Jersey, USA). ELISA Kites were purchased from Elabscience^®^ (Houston, Texas, USA).

###  Screening of Different Essential Natural Oils for the Preparation of NLCs in Combination with the Coconut Oil

 For the preparation of the NLCs, the lipid phase consisted of a mixture of liquid essential oil (eucalyptus oil OR black cumin oil OR tea tree oil) and coconut oil in a ratio of 1:1, which was mixed at a temperature above the coconut oil’s melting point by 10 degrees (≈ 40°C) to ensure complete homogenization. To prepare the medicated formulation, FP was dissolved in the lipid phase to achieve a final concentration of 0.05% (w/w) in the optimized NLC. The aqueous phase consisted of Tween 80, PEG 400, and purified water, heated to the same temperature. The aqueous phase was then added gradually to the lipid phase with ratios as shown in Table 1. Coconut oil was selected as the solid lipid component primarily due to its superior solubilizing capacity for FP (4.2 mg/ml) compared to other screened lipids, which is critical for maximizing drug loading. Furthermore, its high concentration of medium-chain fatty acids (specifically lauric acid) provides inherent skin-penetration-enhancing properties and excellent biocompatibility (GRAS status), making it an ideal carrier for localized psoriatic treatment.^26,27^

**Table 1:** Detailed composition and homogenization times of the different investigated blank NLCs.

**Formulation** **Code**	**Oil phase (gm)**	**Tween 80 (gm)**	**PEG (gm) **	**Distilled water (gm) **	**Homogenization time (min)**
1E (F1)	1	0.25	0.25	8.5	5
2E (F2)	1	0.25	0.25	8.5	10
3E (F3)	1	0.5	0.5	8	5
4E (F4)	1	0.5	0.5	8	10
5B (F5)	1	0.25	0.25	8.5	5
6B (F6)	1	0.25	0.25	8.5	10
7B (F7)	1	0.5	0.5	8	5
8B (F8)	1	0.5	0.5	8	10
9T (F9)	1	0.25	0.25	8.5	5
10T (F10)	1	0.25	0.25	8.5	10
11T (F11)	1	0.5	0.5	8	5
12T (F12)	1	0.5	0.5	8	10

E: Eucalyptus oil, B: Black cumin oil, T: Tea tree oil.

 Afterwards, the formed mixtures were stirred using a magnetic stirrer (DAIHAN Scientific model MSH-20D, Gangwon-DO, South Korea) at 1000 rpm for 5 min to form a coarse pre-emulsion that was further homogenized using high-shear homogenization at 20000 rpm (DAIHAN Scientific model HG 150, Gangwon-DO, South Korea) for one or two 5-minute cycles. The formulation was then incubated for 5 minutes in an ultrasonic bath (Ultrasonicator RoHS cleaner model UD50SH-2LQ, Guangdong Province, China) to remove any formed foam and left standing at 4°C for one hour to stabilize the NLCs before further analysis and characterization.^10,28^

###  Particle Size Analysis and Zeta Potential Determination 

 100 µl of each sample was diluted up to 1 ml using purified water and then vortexed for 30 seconds before measurement. Using a particle size analyzer, dynamic light scattering (DLS) (Zetasizer Nano ZN, Malvern Panalytical Ltd., United Kingdom) at a fixed angle of 173° at 25° C, photon correlation spectroscopy was used to analyze the prepared particles for their size and size distribution in terms of average volume diameters and polydispersity index (PDI). The samples underwent triplicate analysis. Zeta potential was determined using the same apparatus.^29^

###  Entrapment Efficiency Determination

 Encapsulation efficiency was investigated and calculated accordingly using the centrifugation technique, in which drug-loaded NLCs were separated by cooling-centrifuge NEYA16R, Carpi, Italy, at 15000 rpm using a nanosep® centrifuge tube (10 KDa) for 40 minutes to separate the NLCs from the un-entrapped drug. The filtrate was extruded through a 0.2 µm filter and analyzed for its drug content by RP-HPLC.^30^ For the HPLC analysis, an isocratic pump with a flow rate of 1 m/min and equipped with a Waters 996 photodiode array and a Kromasil C18 column (4.6x150 mm (Salisbury, NC, USA)) were utilized along with a water:acetonitrile (40:60 v/v) mobile phase. The entrapment efficiency percentage (EE%) was evaluated by indirect method as shown in Equation (1).^2^


(1) $$EE\%=\frac{Total\;Amount\;of\;FP-Amount\;of\;unentrapped\;FP}{Total\;amount\;of\;FP}\times 100$$

###  Transmission Electron Microscope (TEM)

 A few drops of the NLCs were loaded on a carbon-coated copper grid and left to dry. A 400-mesh copper grid, coated with a thin amorphous carbon film, was used to support the NLC samples for imaging. For staining, a few drops of a freshly prepared solution of phosphotungstic acid were loaded on the grid and left to dry. Then the grid loaded with the sample was examined by HR-TEM (JEOL, JEM-2100, Tokyo, Japan) with a LaB₆ electron source. The measurements were performed at an accelerating voltage of 200 Kv.^31^

###  FTIR Spectroscopy

 Infrared (IR) spectra were recorded on a Fourier-transform infrared (FTIR) spectrometer using the instrument FT-IR Bruker in the frequency range of 400-4000 cm⁻¹ with the resolution of 1 cm⁻¹. FTIR studies were performed for individual drug samples, drug-free NLC formulation, and NLC formulation containing fluticasone; the results were analyzed to investigate the possible interaction.^32^

###  Differential Scanning Calorimetry (DSC)

 The thermal properties of drug-free NLC, FP-loaded NLC, and the free drug were determined by DSC Q2000 V24.4 Bu, Newcastle, Delaware, USA. 5 mg of each sample was placed in hermetically sealed aluminum pans, and a DSC thermogram was obtained at a scanning rate of 5°C/min and heating from room temperature to 350 degree.^33^

###  Ex Vivo Skin Permeation And Deposition

####  Preparation of Skin

 The *ex vivo,* as well as *in vivo,* experiments were approved by the research ethics committee of Ain Shams University (approval number: ENREC – ASU.2020 – 9) and performed in accordance with the national regulations and the Animal Research: Reporting of *In Vivo* Experiments (ARRIVE) guidelines. The National Institutes of Health’s guide for the care and use of laboratory animals (NIH Publications No. 8023, amended 1978) was also followed in the conduct of the study. 200 g male Wistar rats were housed under optimum conditions (25 ± 2°C temperature, 50 ± 10% relative humidity, and a 12 h light/12 h dark cycle). Rats were kept in groups of four per cage and fed a standard diet and water *ad libitum*. The rats were then sacrificed, and the back skin was carefully shaved using electrical clippers. The skin was separated from the subcutaneous fats and cartilages using a scalpel, washed with phosphate-buffered saline, and wrapped in aluminum foil and kept frozen until used.^34^

####  Ex Vivo Skin Permeation 

*Ex-vivo* skin permeation and deposition studies were conducted using Franz diffusion cells equipped with a diffusion area of 1.23 cm^2^ and a receptor compartment volume of 10 ml. The skin samples were carefully mounted with the stratum corneum side facing upwards, and the cells were filled with phosphate-buffered saline (pH 7.4) containing 1% Tween 80 (with 0.42 mg/ml solubility of FP) to maintain proper drug sink conditions. The setup was kept in a thermostatically controlled water bath at 37°C, with the receptor medium constantly stirred at 200 rpm using a Teflon-coated magnetic stir bar. For each experiment, 0.5 g of the tested formulations (FP-loaded NLCs or the commercial Cutivate® cream) was applied to the skin surface. At specified intervals, aliquots were withdrawn from the receptor compartment and replaced with fresh buffer. The concentration of fluticasone propionate in the samples was quantified via HPLC. All experiments were performed in triplicate, and the mean values were reported.^35^

 After internal testing, the chromatographic conditions utilized in this investigation were determined to be exact, dependable, accurate, selective, and sensitive. The linearity of the method was examined for the concentration range of 0.78–25 µg/ml, with a regression coefficient (R^2^) of 0.999, a limit of detection (LOD) of 0.014 µg/ml, and a limit of quantification (LOQ) of 0.042 µg/ml. All method validation findings fell within acceptable ranges, in terms of 0.32% precision (%RSD), 102.42% accuracy (recovery %), 98.5% selectivity, and 95% matrix extraction efficiency.

####  Ex Vivo Skin Deposition

 Skin samples were taken out after the 24-hour skin permeation experiment, and any leftover formulations were cleaned using cotton soaked in phosphate buffer three times. The stratum corneum was then stripped using a tape-stripping technique (stripping for 15 consecutive times), and the dermis and epidermis were separated using forceps to create three separate layers of skin. To guarantee full extraction of any remaining medication, 99.9% ethanol was used to extract each layer, which was then subjected to 30 minutes of sonication. After filtering via 0.2 µm filters, skin extracts were introduced into the HPLC to measure the amount of drug present.^36^

###  In Vivo Evaluation of FP-Loaded NLC Anti-Psoriatic Effect on Imiquimod (IMQ)-Induced Psoriasis In Mice

 In order to test the therapeutic efficacy of optimized FP-loaded black cumin oil NLC (FP-F6 NLC), sixteen C57BL/6 mice were used. C57BL/6 mice have been reported to represent an ideal animal model for psoriasis induction.^37^ The mice were kept at a temperature between 25 and 28 °C, received a standard meal, and had access to fresh water. On day 0 of the investigation, an electrical shaving machine was used to shave 4 cm^2^ of each mouse’s dorsal skin. The experiment was carried out on four groups of mice: Negative control group (normal group, n = 4)—this group did not receive therapy or have psoriasis induced. For the positive control group (model group, n = 4), psoriasis was induced by topical application of IMQ 5% Aldara^®^ cream (83.3 mg/mouse/day) on each mouse’s shaved dorsal area for the first four days, followed by no more treatment. Group A and B (n = 4 for each) were also exposed to a 4-day induction of psoriasis as the model group, followed by treatment of group A *via* topical application with 100 mg Cutivate^®^ cream for 4 consecutive days, while Group B was treated *via* topical application of 100 mg optimized FP-loaded NLC for 4 consecutive days.^38^

####  Psoriasis Area and Severity Index (PASI) Scoring Technique

 Animals were scored using PASI on study days 0, 2, 4, 6, and 8. The parameters to be evaluated were scaling, erythema, and thickness of skin. The scale range for each parameter was 0 to 4, and the grading system was as follows: 0 for none, 1 for slight, 2 for moderate, 3 for marked, and 4 for severe. Two distinct persons calculated the score in order to reduce the likelihood of biased results and take an average. On a scale of 0 to 12, the total PASI was scored, which is the total of the scores for erythema, scales, and average skin thickness.^39^

####  In Vivo Skin Deposition Study

 Animals were put to sleep with an intraperitoneal injection overdose (50–90 mg/kg) of phenobarbital one hour after the final topical treatment dose was applied, and they were then euthanized. The amount of drug deposited in the skin was measured by removing treated skin sections and cleaning them with normal saline. The shredded skin was then combined with 10 ml ethanol and stirred on a magnetic stirrer for 30 minutes to extract the drug that was retained. The resulting mixture was then filtered through a 0.2 μm filter and injected into an HPLC to measure the amount of drug deposited in the skin.^40^

####  Histopathological Analysis

 Autopsy dorsal skin samples from mice in different groups were collected and preserved for 24 h in 10% paraformaldehyde saline. Samples were first rinsed by distilled water before being dehydrated by serial concentrations of alcohol (methyl, ethyl, and absolute ethyl, respectively). After that, they are cleaned with xylene and fully covered with paraffin for 24 hours at 56°C in a hot air oven. Using a slide microtome, paraffin tissue blocks were created for sectioning at a thickness of 5 μm. The following steps include collection on glass slides, deparaffinization, and finally staining by hematoxylin and eosin for histological examination.^39^

####  Evaluation of Cytokine Secretions

 Topical application of IMQ cream on mouse skin results in excessive stimulation of keratinocytes and increased production of cytokines involved in the pathogenesis of psoriasis.^41^ Enzyme-linked immunosorbent assay (ELISA) was used to measure the levels of IL-1α, IL-6, and IL-17a in the skin extracts. Skin samples were properly cleansed in ice-cold PBS to eliminate any residual blood, and they were weighed before being homogenized. Using a glass homogenizer on ice (micro tissue grinder) and lysis buffer, tissues were minced into minute pieces. An ultrasonic cell disrupter was used to sonicate the resultant suspension until complete homogenization. The homogenates were then centrifuged at 10,000 g for 5 min. The supernatant was taken, and the assay was carried out according to the manufacturer’s instructions of the used ELISA kits.^42^

###  Statistical Analysis

 GraphPad Prism version 8 software (GraphPad Software, San Diego, CA) was used to perform the statistical analysis. The level of statistical significance was determined by analysis of variance (ANOVA) followed by Tukey’s test for multiple comparisons, where *P* < 0.05 was considered statistically significant.^43^

## Results and discussion

###  Selection of Liquid Oil Component of NLCs

 Values of particle size, PDI, and zeta potential of the prepared blank NLC formulations are shown in Table 2, and results were used to determine the best formulation produced.

**Table 2:** Particle size, PDI, and zeta potential values (n = 3 ± SD) of NLC formulations.

**Formulation**	**Particle size (nm)**	**PDI**	**Zeta potential (mV)**
1E (F1)	555.2 ± 99.15	0.98 ± 0.01	-12.2 ± 0.35
2E (F2)	175.9 ± 4.54	0.64 ± 0.01	-13.2 ± 0.61
3E (F3)	Phase separation		
4E (F4)	Phase separation		
5B (F5)	207.3 ± 2.05	0.44 ± 0.01	-29 ± 0.76
6B (F6)	151.5 ± 2.15	0.4 ± 0.02	-19.1 ± 0.12
7B (F7)	Phase separation		
8B (F8)	176.2 ± 3.2	0.45 ± 0.02	-20.6 ± 0.5
9T (F9)	224.2 ± 5.98	0.79 ± 0.06	-10.2 ± 0.33
10T (F10)	146.6 ± 2.78	0.59 ± 0.05	-11.2 ± 0.61
11T (F11)	Phase separations		
12T (F12)	Phase separation		

 As shown in Table 2, both F5 and F6 have the most appropriate results regarding both low particle size and polydispersity index. It can be attributed to increased surface area by the action of optimum concentrations of Tween 80 and PEG 400, where they resulted in a high negative charge and repulsive forces between particles, providing stable, homogenous particles.^13^

 Both formulations 5B (F5) and 6B (F6) had the same compositions but different homogenization times. The longer homogenization time led to a significant reduction in the particle size and homogeneity of the particles, and F6 was the only formula with a PDI value lower than 0.4. Therefore, F6 was selected for further drug loading and characterization based on the holistic consideration of particle size, zeta potential, PDI, and the potential for effective drug loading.

###  Characterization of the Selected FP-Loaded NLC Formulation

####  Particle Size, PDI, and Zeta Potential Determination

 Results showed the particle size of selected FP-F6 NLC was 171.3 ± 1.99 nm with PDI 0.37 ± 0.01, which indicated an optimum particle size suitable as a topical drug delivery system,^44^ and low PDI, which indicated that the formulation was homogenous. Zeta potential was -32.3 ± 0.5 mV, which indicated a stable formulation due to repulsive forces between particles; also, Tween 80 provided steric stabilization between particles, preventing any agglomeration, and the negative charge is attributed to oxygen atoms in the PEG 400 molecules.^45^

####  Entrapment Efficiency 

 The drug entrapment efficiency within the NLC was found to be 46.01% ± 1.12. FP has fairly good solubility in the surfactants and solubilizers used. As FP solubilities in Tween 80 and PEG 400 are 4.67 mg/ml and 4.33 mg/ml respectively,^46^ while saturation solubility of FP studies of our group in natural essential oils showed it is 0.13 mg/ml in black cumin oil and 4.2 mg/ml in coconut oil. So this entrapment efficiency ratio resulted from the partitioning of FP between the oil phase and the aqueous phase, which tended to pull FP out of the oil phase.^47^

 This partitioning is typically intensified during high-energy hot homogenization, where elevated temperatures further increase the solubility of lipophilic FP within surfactant micelles, facilitating an escaping phenomenon from the molten lipid droplets before solidification.^48^

 Furthermore, the incorporation of bioactive black cumin oil creates a complex, disordered lipid lattice. The competition for space between FP and the various fatty acid constituents of the natural oil may further limit the capacity of the lipid matrix to accommodate the drug compared to simpler lipid systems.^48,49^

###  Transmission Electron Microscope (TEM)

 Transmission electron microscopic images of NLC showed spherical-shaped particles as shown in Figure 1, and particle size range distribution matched with results obtained from dynamic light scattering analysis. Particles were less than 200 nm, a size particularly suitable for topical delivery.^50^

**Figure 1:**
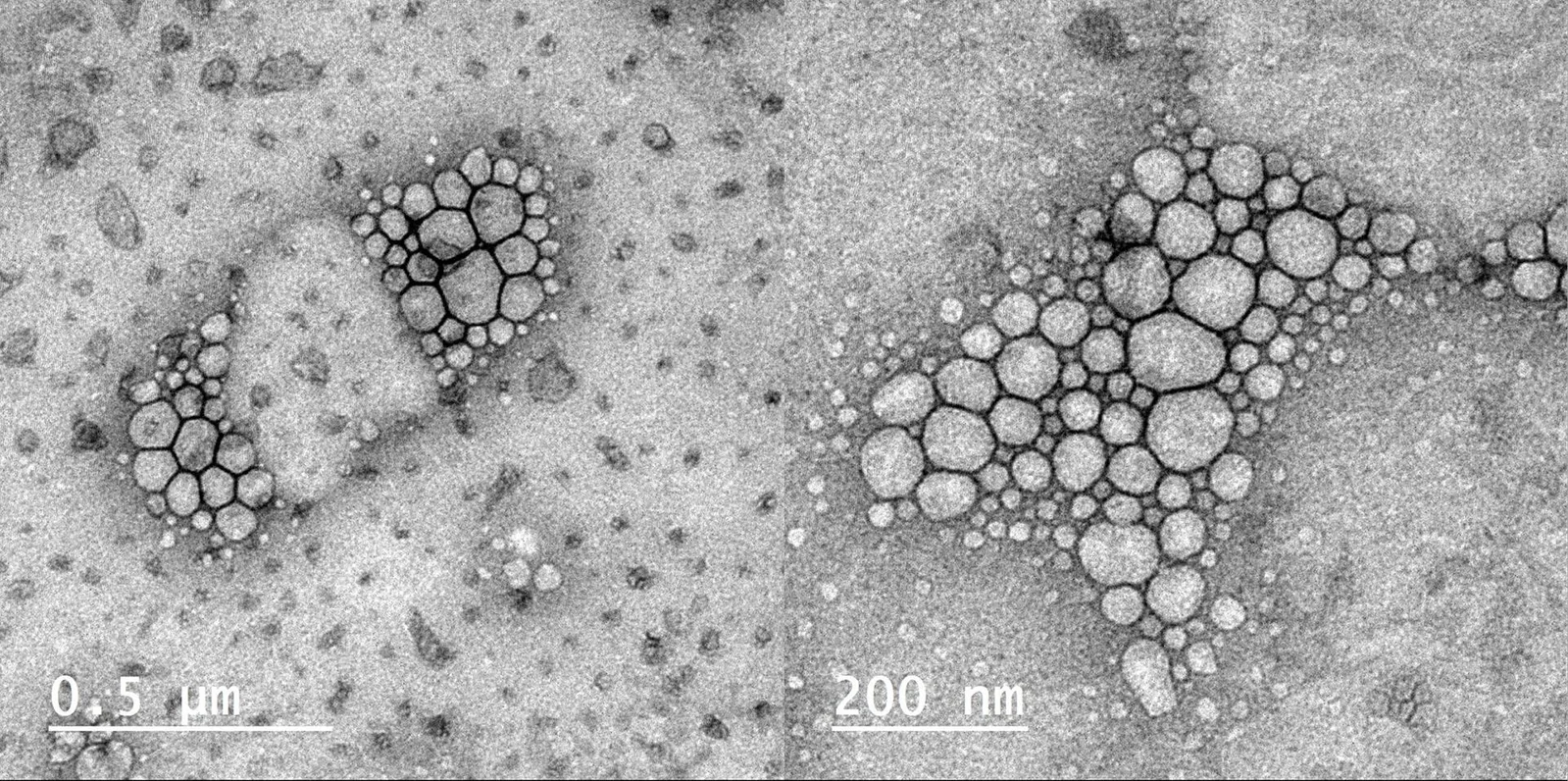
Morphological aspects of NLC displayed by TEM

###  FTIR Spectroscopy

 In order to investigate interactions between drugs and excipients in formulation. The samples’ measurements of pure drug, drug-free NLC, and FP-loaded NLC were done from the range of 500 to 4000 cm⁻¹ with a resolution of 1 cm⁻¹ (Figure 2).

**Figure 2:**
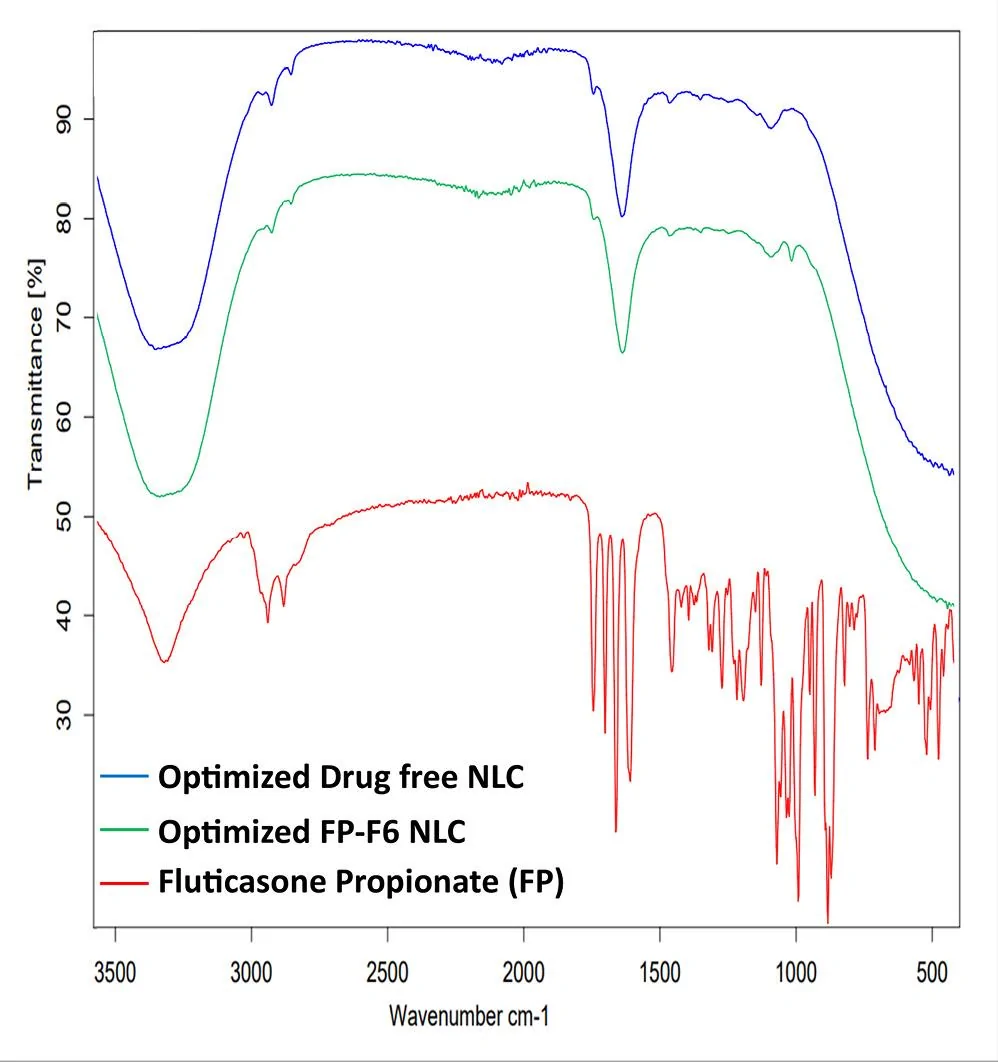
IR spectrum of the pure FP, optimized drug-free NLC, and the optimized FP-F6 NLC

 From the FT-IR spectra of the stable nature of FP, the principle peaks are obtained at the following wavelengths: 1454.72 cm⁻¹ (-OH) stretch for the hydroxyl group, 990.36 cm⁻¹ (S-H) thiol stretch for the thiol group, 882.28 cm⁻¹ (C-O) stretch for the ether group, 929.67 cm⁻¹ (C-H) bend for the 5-item ring, 735.84 cm⁻¹ (C-H) bend for the aromatic benzene ring, and 2938.64 cm⁻¹ (C-H) stretch for aldehyde.

 In the crystalline fluticasone propionate, the stretching of the -OH peak at 3318.57 cm⁻¹ suggested an external hydrogen bonding. The carbonyl functional group (C = O) linked to the aliphatic ring is responsible for the peak at 1743 cm⁻¹, while the carbonyl group coupled to sulfur (S-C = O) displayed a peak at 1,699.89 cm^−1^. The carbonyl group (C = O) was found to be stretched at a peak of 1,659.92 cm^−1^, whereas the quinonoid aromatic ring’s vibrational stretching was seen at 1,607.8 cm^−1^. The stretching vibrations peak for F, C, and S was measured at 1033.75 cm⁻¹, and the peak for C–F stretching vibrations was seen at 1270.61 cm⁻¹.^51,52^ The IR spectra of NLC free-drug formulation and drug-loaded NLC are similar, and the characteristic peaks of pure FP have disappeared, reflecting that lipid forms the outer core and the drug is incorporated inside it, and there is no drug on the surface.^53^

###  Differential Scanning Calorimetry (DSC)

 Pure FP is decomposed by a small endothermic peak at 271.72°C, and this peak indicates its crystalline structure.^54^ Thermogram of both optimized free drug formulation and loaded drug formulation showed endothermic peaks at 112.8°C and 111.76°C, respectively. There was no significant effect of FP incorporation on lipid matrix crystallinity and melting peaks, and results were matched with Doktorovová et al.^55^ The peak of FP disappeared, indicating that the crystal structure of FP was overcome by the amorphous structure of the NLC lipid matrix (Figure 3).

**Figure 3:**
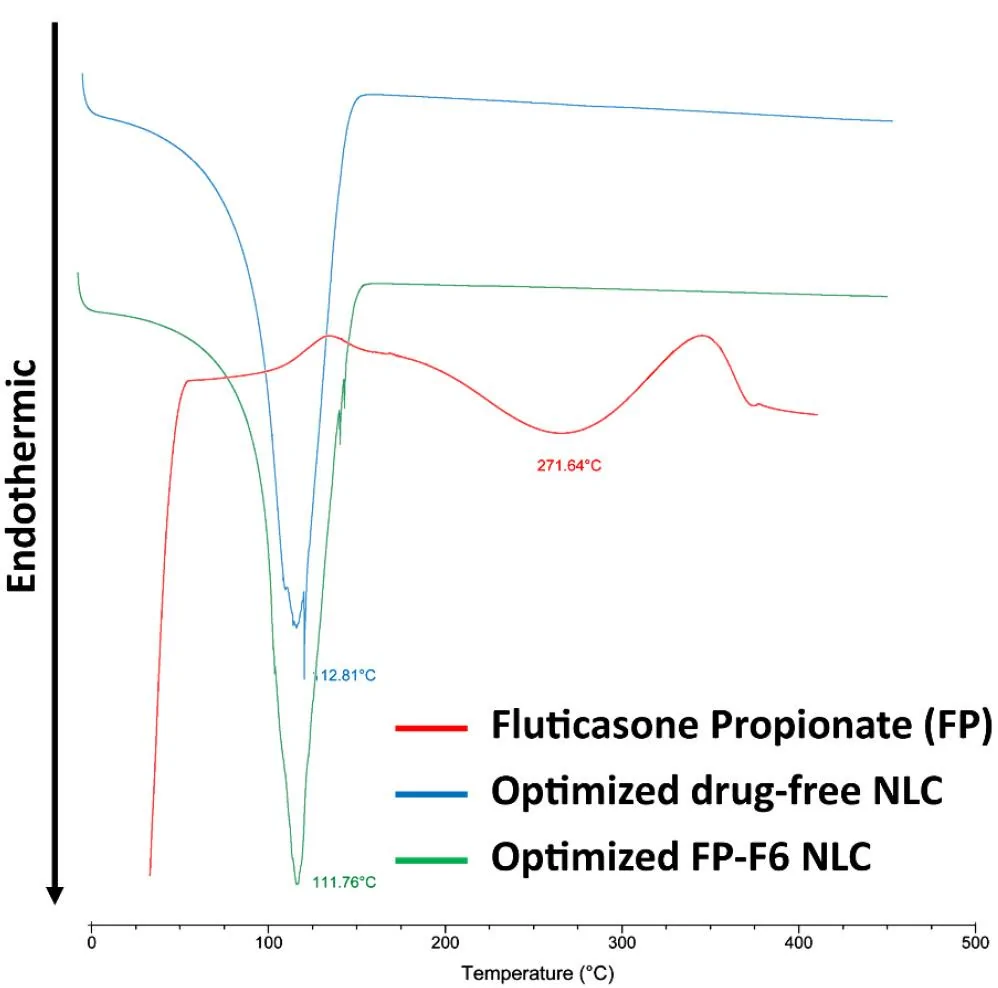
DSC of pure drug, optimized drug-free NLC, and optimized FP-F6 NLC

###  Ex Vivo Skin Permeation And Deposition

 Generally, many factors participate in making NLC act as a permeation enhancer for drugs. Factors include both the ability of NLC to interact with the skin surface and the ability to form a hydrophobic film, which helps increase skin moisture, resulting in increased gaps between corneocytes and, therefore, enhanced permeation. In addition to the presence of surfactant affecting skin layers and fluidity, there is an intercalating effect of Tween 80 (polysorbate 80). It has a chain of 18 carbons with cis unsaturation, and this intercalating effect causes lipid disorder, leading to higher permeation ability through skin compared to the market cream Cutivate®, where no drug was detected in the receptor compartment (Figure 4a).^56^ To show the permeation-enhancing effect of NLC, the steady-state flux value (*J*_ss_) and permeability coefficient (*P*_c_) were calculated, which were 4.08 × 10⁻⁵(µg cm⁻^2^ h⁻¹) and 2.09 × 10⁻⁷(cm h⁻¹), respectively. These findings indicated the superior permeability-enhancing effect of the optimized FP-F6 NLC formulation. NLC ensures covering the skin surface, preventing moisture evaporation and increasing stratum corneum thickness, allowing enhanced drug permeation.^11^

**Figure 4:**
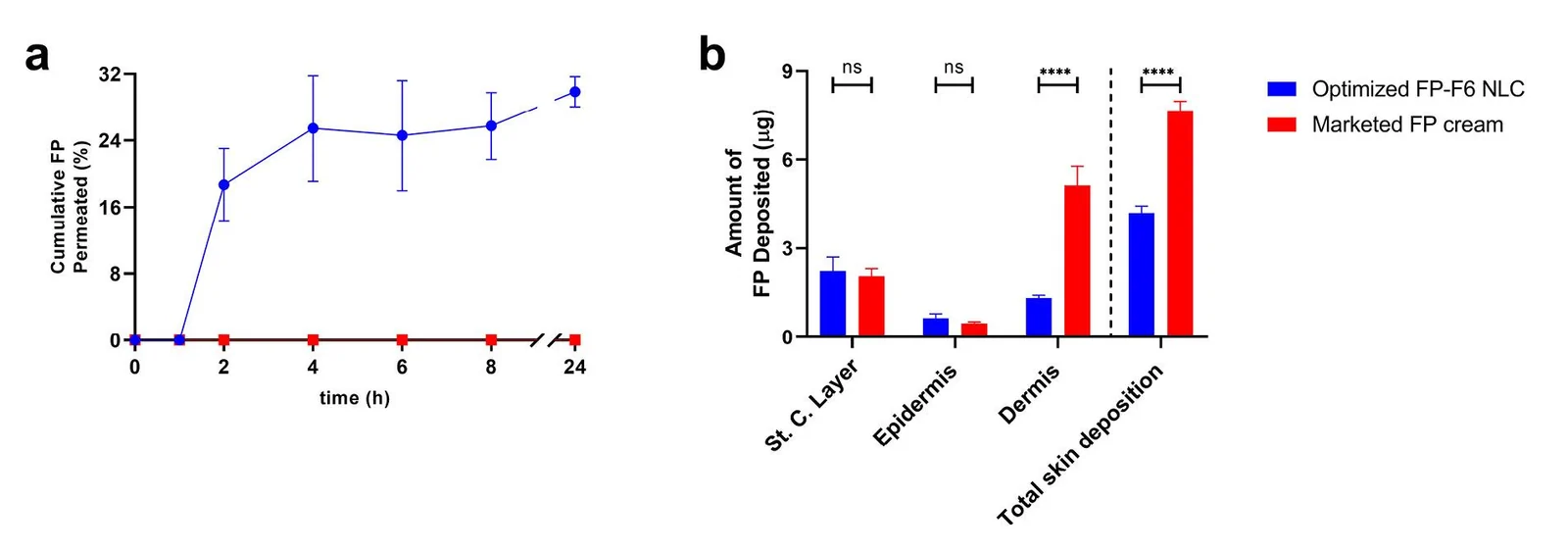
Cumulative percentage of FP permeated from the FP-F6 NLC formulation (a), and amount of skin deposited FP of FP-F6 NLC and marketed FP cream in different skin layers (b). Data are expressed as mean ± SD (n = 3). *, **, ***, and **** denote significant difference at *P* < 0.05, *P* < 0.01, *P* < 0.001, and *P* < 0.0001, respectively, while ns denotes the non-significant difference of data

 Regarding skin deposition, the optimized FP-F6 NLC achieved a skin deposition of 2.24 ± 0.46 µg of the applied drug in the SC layer, the target site to control psoriatic plaques, compared to 2.06 ± 0.25 µg after application of marketed cream.^57^ SC acts as a barrier for drug delivery exaggerated by thickening and hyperkeratosis, in addition to the pathology of psoriasis originating from the epidermal layer as excessive skin hyperproliferation occurs.^58^ Deposited FP in the epidermal layer after topical application of optimized FP-F6 NLC was 0.64 ± 0.15 µg compared to 0.47 ± 0.05 µg after application of marketed cream (Figure 4b), while in the dermis layer, the optimized formulation achieved a skin deposition of 1.32 ± 0.1 µg compared to 5.13 ± 0.65 µg after application of marketed cream, which means that the NLCs achieved a total skin deposition less than the marketed product.^59^ While the statistical analysis shows no significant difference in the amount of FP deposited in the upper two layers between the optimized FP-F6 NLC and the marketed cream, the preferential distribution of the optimized FP-F6 NLC formulation within the stratum corneum and epidermis skin layers, in contrast to the marketed cream’s higher deposition in the dermis layer over other skin layers, is the key to treating psoriasis. This targeted deposition is more therapeutically relevant for psoriasis management, as the disease primarily affects these upper layers. Therefore, while the overall deposition of NLC formulation is lower than the marketed cream (mainly owing to the permeated portion of FP-F6 NLC), the enhanced concentration in these specific layers with the presence of the different oils used with their inherent anti-inflammatory activity, higher *in vivo* efficacy can be expected.

###  In Vivo Evaluation of FP-Loaded NLC Anti-Psoriatic Effect on Imiquimod (IMQ)-Induced Animal Psoriasis

 The IMQ mouse model was constructed in three groups: the positive control (model) group, group A, and group B. Skin thickness, erythema, and scales gradually increased after daily application of IMQ cream for 4 days, but minimal scales and no erythema were visible on the first two days. As the third and fourth days progressed, the level of scaling and erythema increased. Figure S1 illustrated this progress in manifestations. Treatment started from day 5 using a marketed cream (group A) vs optimized FP-F6 NLC (group B). The findings revealed that all manifestations were improved in the treatment group B, and the PASI score declined rapidly. There was a complete disappearance of scales in most animals of group (B) to a greater extent than in group A.

 The sign of significant healing of inflamed skin is the partial return to normal skin color and thickness (Figure S2). The total PASI score increased from zero on the first day to 10.75 ± 0.96 on the fourth day in the model (IMQ psoriasis-induced groups), which indicated the success of the construction of the psoriasis model (Figure 5).

**Figure 5:**
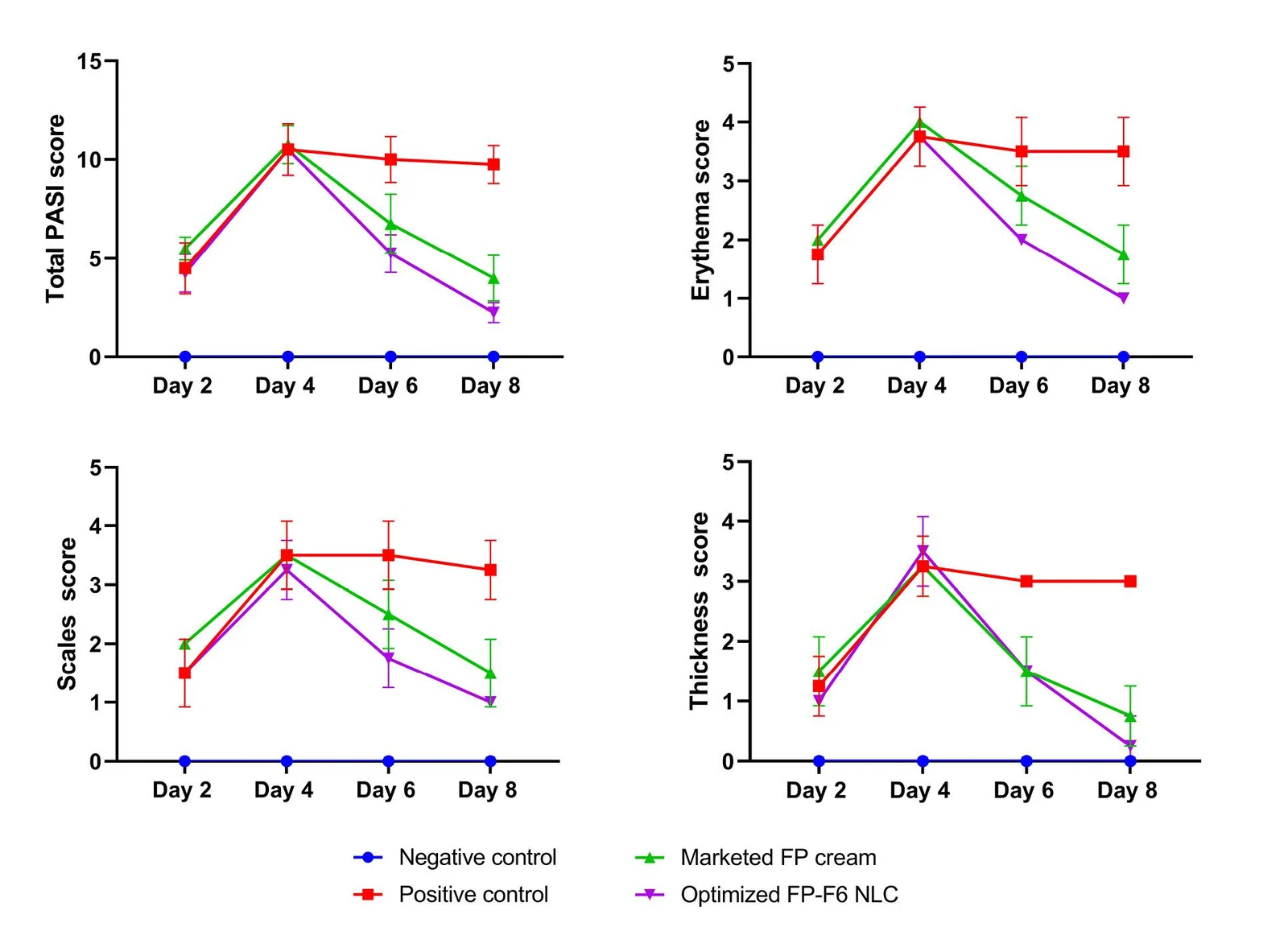
Psoriasis assessment parameters and total psoriasis area severity index score for the negative control (normal) group, positive control (model) group, group A (treated with marketed FP Cutivate® cream), and group B (treated with the optimized FP-F6 NLC). Results are displayed as mean values ± SD (n = 4)

 The PASI score decreased significantly in the FP NLC group with a *P* value < 0.0001 compared to the positive control group. These results showed the marked curative effect of the optimized formulation regarding alleviation of psoriasis symptoms. Additionally, the PASI score decreased upon application of treatment in group A (Cutivate® cream). The progress of scores of scaling, erythema, and thickness in treated groups can be observed separately (Figure 5). Regarding dorsal skin thickness, both optimized FP-F6 NLC and the marketed cream showed a decline in score after day 2 of starting treatment application. Healing in the group treated with optimized FP-F6 NLC was faster than in the marketed group with *P* < 0.0001, reaching an average score of 0.25 at the end of treatment in the optimized formulation, which was nearly the same skin thickness as day 0, while the score was 1.75 in the marketed cream group. For erythema score, the optimized FP-F6 NLC showed significant reduction of erythema score (*P* < 0.002) versus the marketed cream, as it reached an average score of 1 for the optimized formulation group compared to 1.75 for the marketed cream. As for scaling score, optimized FP-F6 NLC showed significant improvement in scales (*P* < 0.0001) compared to the marketed cream, reaching an average score of 1.75 on day 6 and 1 on day 8, while the marketed cream group’s average score was 2.75 on day 6 and 1.75 on day 8. The revealed superior improvement in the group treated with the optimized FP-F6 NLC is potentially referred to the combinational influence of the strong anti-inflammatory effect of FP alongside the black cumin used in the NLCs’ formulation, which proved to have significant anti-psoriatic efficacy as reported in an earlier study.^60^ It was also noted that hair growth was remarkable in the optimized FP-F6 NLC-treated group, which signifies a healthy sign of the skin along with a remarkable reduction of inflammation.^38,39^ Beyond acting as a structural solid lipid matrix, coconut oil’s high concentration of medium-chain fatty acids exerts a profound emollient effect (as mentioned in Section 2.2). This directly improves stratum corneum hydration, reinforces the skin’s lipid barrier, and upregulates essential barrier proteins (like filaggrin and involucrin), thereby reducing the transepidermal water loss (TEWL) characteristic of compromised psoriatic plaques.^61^

 The total amount of deposited FP in different skin layers is an important factor to study the efficacy of the optimized NLC formulation. Upon its measurement, it was found that results were matched with the *ex vivo* study. The amount of the deposited FP in group (B) (treated with the optimized FP-F6 NLC formulation) was 0.036 ± 0.013 μg/mg of excised skin, compared to group A treated with the marketed cream (0.0202 ± 0.01 μg/mg of excised skin), where no significant difference was detected, but as expected, the therapeutic efficacy was higher in the case of the NLCs.^62^

###  Histopathological Analysis

 Following imiquimod administration, rete ridge elongation with hyperkeratosis and parakeratosis, vascular dilatation, and inflammatory cell infiltration were noticed in the dermis (Figure 6). The photomicrograph of skin for the optimized FP-F6 NLC group showed the highest improvement, with partial increasing thickness of the epidermal layer and congestion of dermal blood vessels.

**Figure 6:**
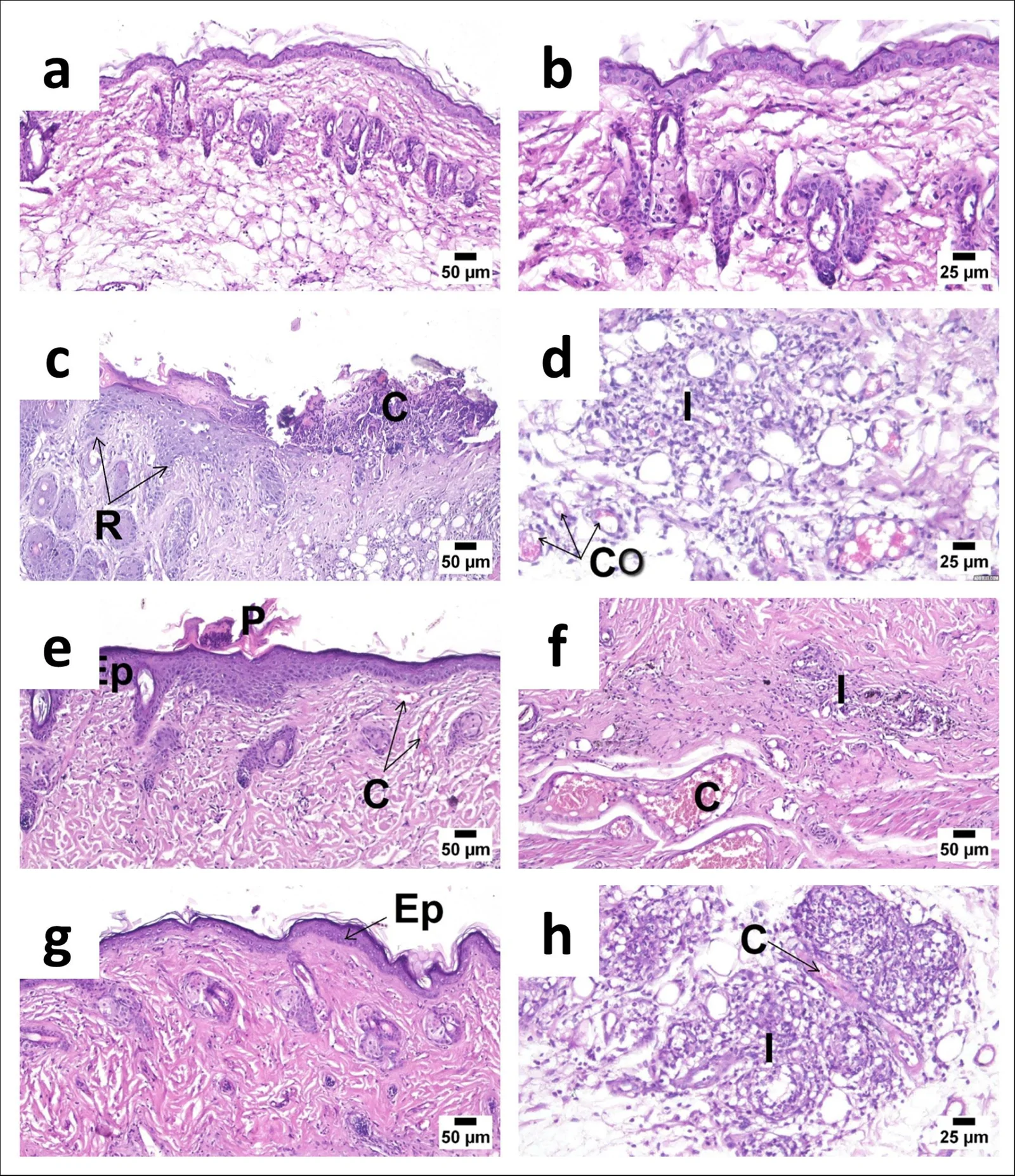
Photomicrographs of mice’s skin stained with Hematoxylin and Eosin (H&E).Photomicrographs of skin from the normal group (a and b) showed normal histological structure of skin, while photomicrographs of skin from the model group (c and d) displayed an increase in thickness of epidermal layer (Ep) with formation of rete ridges (R), presence of ulcer covered by serocellular crust (C), dermal infiltration by mononuclear inflammatory cells (I), and congestion of dermal blood vessels (CO). Besides, photomicrographs of skin obtained from the optimized FP-F6 NLC group (e and f) showed partial increasing in thickness of epidermal layer (Ep), partial dermal infiltration by mononuclear inflammatory cells (I), parakeratosis formation (P), and congestion of dermal blood vessels (C). Moreover, a photomicrograph of skin from the marketed FP cream group (g and h) shows a partial increase in thickness of the epidermal layer (Ep) and congestion of dermal blood vessels (C) with dermal infiltration by mononuclear inflammatory cells (I)

 Epidermal thickness was measured in skin specimens by using image analysis software (ImageJ 1.47v, NIH, USA), and results are shown in Figure 7.^63^ The outcomes revealed that there was a significant difference in epidermal thickening between the model positive group and the optimized FP-F6 NLC-treated group or the one treated with the market cream (*P*-value < 0.0001), indicating the efficacy of both systems to alleviate the symptoms.

**Figure 7:**
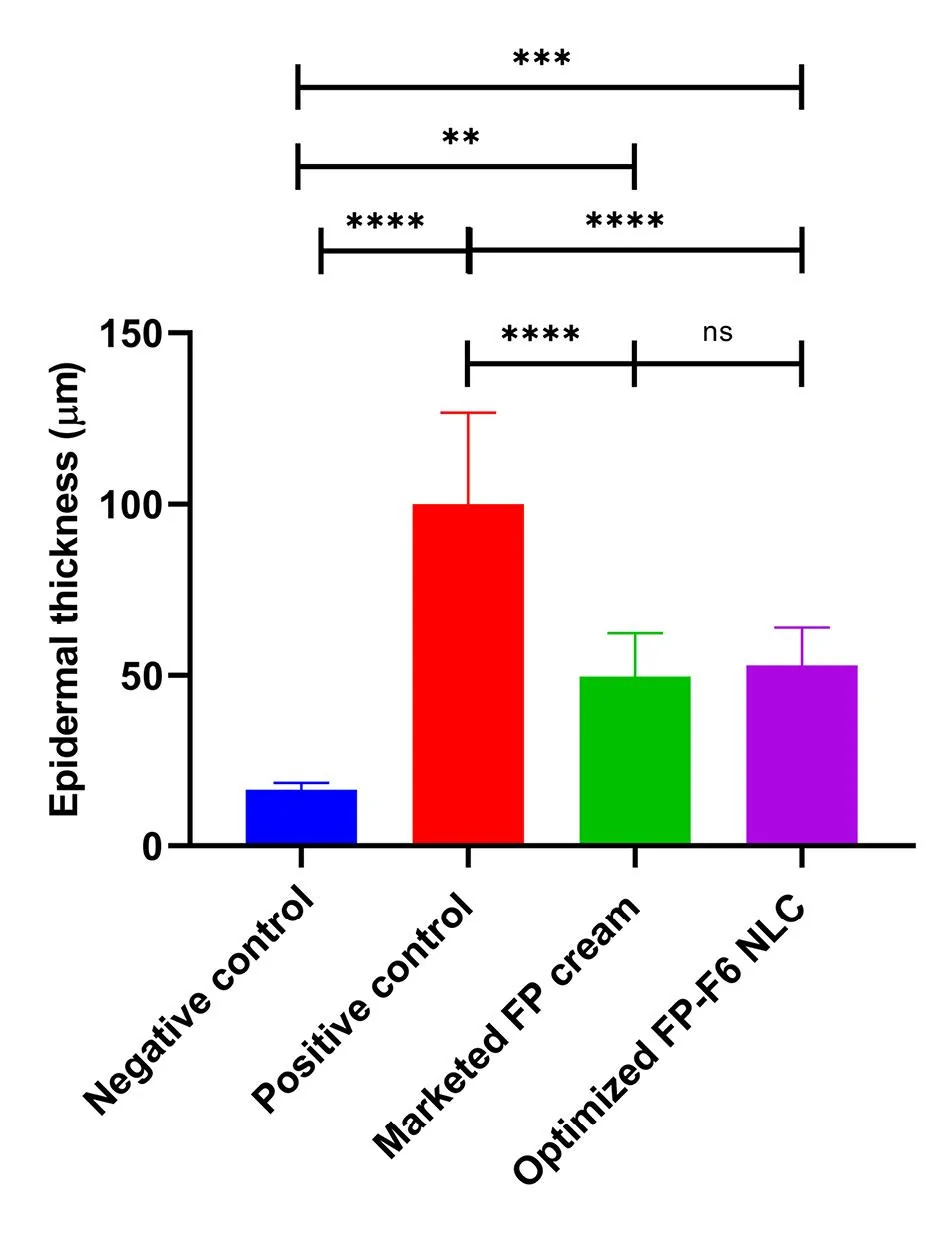
Epidermal thickness of negative control, positive control, marketed FP cream group (A), and optimized FP-F6 NLC group (B). (n = 4). *, **, ***, and **** denote significant difference at *P* < 0.05, *P* < 0.01, *P* < 0.001, and *P* < 0.0001, respectively, while ns denotes the non-significant difference of data

###  Evaluation of Cytokine Secretions

 IL-1α, IL-6, and IL-17a are cytokines that play a major role in the pathogenesis of psoriasis and the IL-17/IL-23 axis. Cytokines increase significantly after application of IMQ cream and construction of the model. Thus, measurement of their levels in skin samples is an indicative factor to assess the prognosis of disease and the efficacy of different applied formulations.^64^

 Upon measurement of the level of IL-1α of the excised skin samples to determine antipsoriatic activity,^65^ the level of IL-1α in group B treated with the optimized FP–F6 NLC formulation was found to be 97.13 ± 6 pg/mg (Figure 8), which indicated a reduced level of interleukin that significantly differed (*P*-value < 0.0001) from the level observed in the positive control model group (259.3 ± 7.6 pg/mg). In addition, there was a significant difference between the marketed cream and the optimized FP–F6NLC formulation in reducing the level of IL-1α (*P*-value = 0.0027), which confirmed the better performance of the optimized NLC formulation and effective curative anti-inflammatory effect of natural excipient oils black cumin oil and coconut oil.

**Figure 8:**
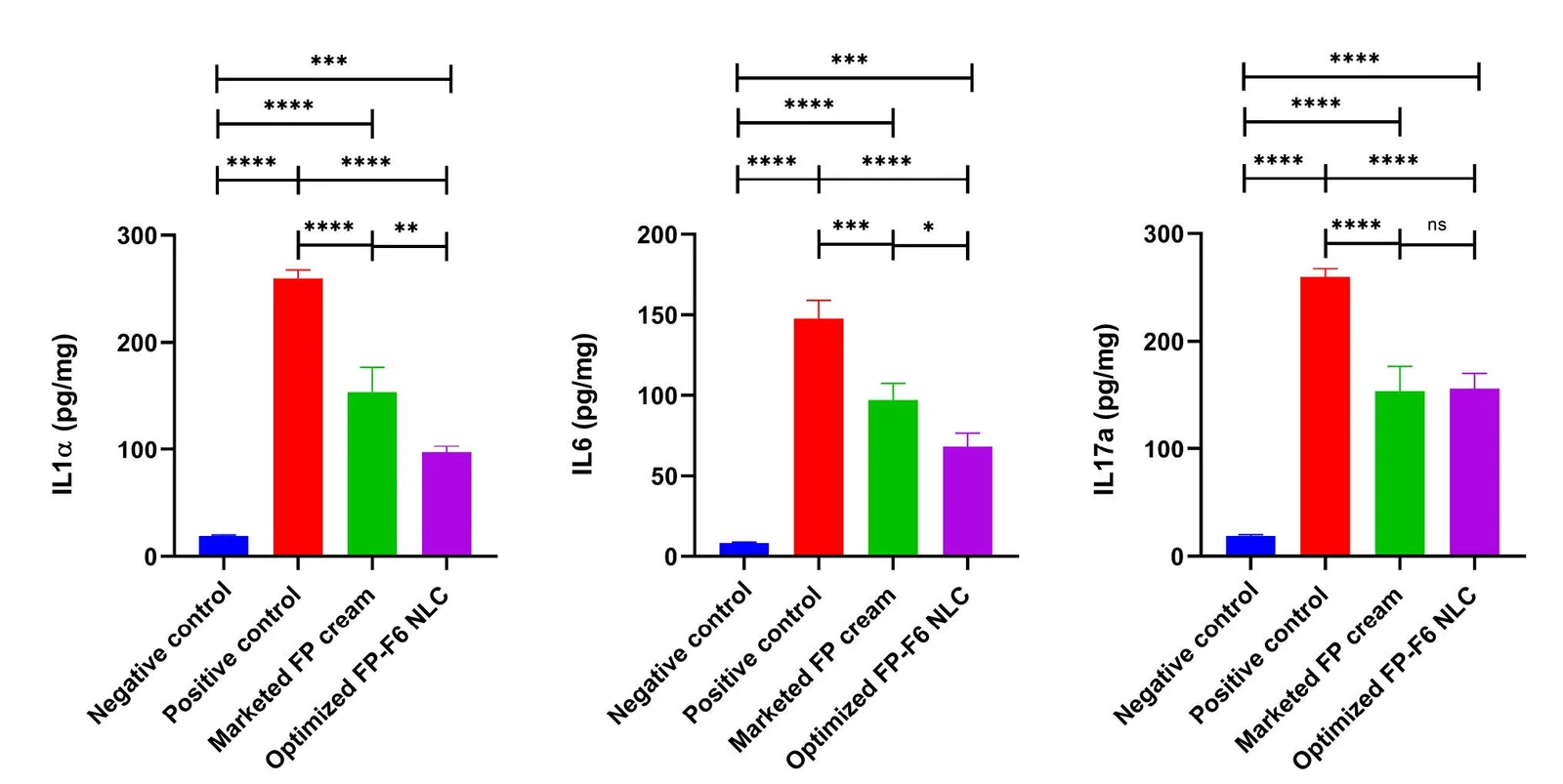
Levels of IL-1α, IL6, and IL17a in all experimental groups (n = 4). *, **, ***, and **** denote significant difference at *P* < 0.05, *P* < 0.01, *P* < 0.001, and *P* < 0.0001, respectively, while ns denotes the non-significant difference of data

 The level of IL-6 in group B treated with the optimized FP–F6 NLC formulation was 68.39 ± 8.38 pg/mg, which also indicated a reduced level of interleukin with a significant difference (*P*-value < 0.0001) from the level of IL-6 in the positive control model group (147.7 ± 11.1 pg/mg). Also, there was a significant difference between the marketed FP cream and the optimized FP–F6 NLC formulation in reducing IL-6 level (*P*-value = 0.0158), which ensured the enhanced healing performance of the optimized formulation.

 Regarding IL-17a, there was a significant reduction in IL-17a levels of optimized FP-F6 NLC (155.93 ± 14 pg/mg) compared to the model control (282.83 ± 22.03 pg/mg) (*P*-value < 0.001) (Figure 8).^66^

 In a nutshell, all the aforementioned outcomes, including the visual inspection, PASI score, *in vivo* skin deposition, and the histopathological manifestation, confirmed the superiority of the optimized FP-F6 NLC formulation as an effective anti-psoriatic weapon. The optimized formulation offered a promising approach in maximizing efficacy and optimizing skin deposition in the targeted skin layers with minimal systemic side effects.

## Conclusion

 It can be concluded that the optimized FP–loaded black cumin oil NLC formulation was optimized for various factors. The formulation was engineered by the hot melt homogenization method, which proved to be a successful approach that provided an optimized safe formulation with low particle size and PDI without the use of organic solvent for maximum benefit of incorporation of FP. In addition, the displayed *ex vivo* drug skin deposition and permeation profile rendered the topical application of the optimized FP-loaded black cumin oil NLC with better pharmacodynamic behavior, which confirmed the achieved synergistic action between FP and the anti-inflammatory effect of black cumin oil for managing psoriasis. This was clearly reflected in the reduced PASI score and inflammatory interleukins involved in psoriasis alongside the improved histological manifestation in the IMQ-induced psoriasis model compared to the marketed FP cream. To conclude, the combinatorial activity of the optimized FP-loaded black cumin oil NLC has gained a better overall curative profile over the marketed FP cream. We acknowledge the limitation regarding the sample size (n = 4). While this cohort size was sufficient to demonstrate statistically significant differences in PASI scores, histological thickness, and cytokine levels, we recognize that the statistical power is inherently limited. This study was designed as a preliminary investigation into the efficacy of the NLC system, and future studies with larger cohorts will be considered to further validate these findings and enhance statistical robustness. As a future perspective, the optimized formulation can be considered for scaling up and for human clinical trials, taking into consideration potential future studies to investigate the correlation between NLC surface and interface characteristics and their dual deposition/permeation performance in treating psoriatic plaques via a Quality by Design approach.

## Availability of Data and Material

 All data generated or analyzed during this study are included in this article.

## Competing Interests

 The authors declare that they have no competing interests.

## Declaration of Generative AI and AI-Assisted Technologies in the Writing Process

 The authors of this research work utilized QuillBot/Paraphraser (standard/free version) as little as possible to enhance language and readability. The authors took full responsibility for the publication’s content and also confirmed that they have reviewed and edited the content thoroughly after using this tool/service.

## Ethical Approval

 The experiment was approved by the research ethics committee of Ain Shams University (approval number: ENREC–ASU.2020–9) and performed in accordance with the national regulations and the Animal Research: Reporting of *In Vivo* Experiments (ARRIVE) guidelines. The National Institutes of Health’s guide for the care and use of laboratory animals (NIH Publications No. 8023, amended 1978) was also followed in the conduct of the study.

## Supplementary Files

Supplementary Materials. FP-loaded Black Cumin Oil NLC for Psoriasis

## References

[1] Singhvi G, Hejmady S, Rapalli VK, Dubey SK, Dubey S. Nanocarriers for topical delivery in psoriasis. In: Shegokar R, ed. Delivery of Drugs. Elsevier; 2020. p. 75-96. doi: 10.1016/b978-0-12-817776-1.00004-3.

[2] Fereig SA, El-Zaafarany GM, Arafa MG, Abdel-Mottaleb MM (2021). Self-assembled tacrolimus-loaded lecithin-chitosan hybrid nanoparticles for in vivo management of psoriasis. Int J Pharm.

[3] Glickman FS (1986). Lepra, psora, psoriasis. J Am Acad Dermatol.

[4] Petit RG, Cano A, Ortiz A, Espina M, Prat J, Muñoz M (2021). Psoriasis: from pathogenesis to pharmacological and nano-technological-based therapeutics. Int J Mol Sci.

[5] Ramanunny AK, Wadhwa S, Singh SK, Sharma DS, Khursheed R, Awasthi A (2020). Treatment strategies against psoriasis: principle, perspectives and practices. Curr Drug Deliv.

[6] Raharja A, Mahil SK, Barker JN (2021). Psoriasis: a brief overview. Clin Med (Lond).

[7] Murphy EC, Schaffter SW, Friedman AJ (2019). Nanotechnology for psoriasis therapy. Curr Dermatol Rep.

[8] Pradhan M, Singh D, Singh MR (2013). Novel colloidal carriers for psoriasis: current issues, mechanistic insight and novel delivery approaches. J Control Release.

[9] Mendes IT, Ruela ALM, Carvalho FC, Freitas JT, Bonfilio R, Pereira GR (2019). Development and characterization of nanostructured lipid carrier-based gels for the transdermal delivery of donepezil. Colloids Surf B Biointerfaces.

[10] Sathe P, Saka R, Kommineni N, Raza K, Khan W (2019). Dithranol-loaded nanostructured lipid carrier-based gel ameliorate psoriasis in imiquimod-induced mice psoriatic plaque model. Drug Dev Ind Pharm.

[11] Keshri L, Pathak K (2013). Development of thermodynamically stable nanostructured lipid carrier system using central composite design for zero order permeation of econazole nitrate through epidermis. Pharm Dev Technol.

[12] Okonogi S, Riangjanapatee P (2015). Physicochemical characterization of lycopene-loaded nanostructured lipid carrier formulations for topical administration. Int J Pharm.

[13] Kaur N, Sharma K, Bedi N (2018). Topical nanostructured lipid carrier-based hydrogel of mometasone furoate for the treatment of psoriasis. Pharm Nanotechnol.

[14] Dwarampudi LP, Palaniswamy D, Nithyanantham M, Raghu PS (2012). Antipsoriatic activity and cytotoxicity of ethanolic extract of Nigella sativa seeds. Pharmacogn Mag.

[15] Okasha EF, Bayomy NA, Abdelaziz EZ (2018). Effect of topical application of black seed oil on imiquimod-induced psoriasis-like lesions in the thin skin of adult male albino rats. Anat Rec (Hoboken).

[16] Ahmed JH, Ibraheem AY, Al-Hamdi KI (2014). Evaluation of efficacy, safety and antioxidant effect of Nigella sativa in patients with psoriasis: a randomized clinical trial. J Clin Exp Investig.

[17] Majdalawieh AF, Fayyad MW (2015). Immunomodulatory and anti-inflammatory action of Nigella sativa and thymoquinone: a comprehensive review. Int Immunopharmacol.

[18] Sahu S, Katiyar SS, Kushwah V, Jain S (2018). Active natural oil-based nanoemulsion containing tacrolimus for synergistic antipsoriatic efficacy. Nanomedicine (Lond).

[19] Ashraf M, El-Sawy HS, El Zaafarany GM, Abdel-Mottaleb MM (2023). Can essential oils/botanical agents smart-nanoformulations be the winning cards against psoriasis?. Pharmaceutics.

[20] Ashraf M, El-Sawy HS, El Zaafarany GM, Abdel-Mottaleb MM (2025). Eucalyptus oil nanoemulsion for enhanced skin deposition of fluticasone propionate in psoriatic plaques: a combinatorial anti-inflammatory effect to suppress implicated cytokines. Arch Pharm (Weinheim).

[21] Trismayanti NMR, Handono K, Handayani D, Kalsum U, Rukmigarsari E (2024). Pathway analysis of the effect of virgin coconut oil on suppressing colonic mucosal inflammation and clinical symptoms in ulcerative colitis. Pharmacia.

[22] Prabahar K, Udhumansha U, Elsherbiny N, Qushawy M (2022). Microneedle mediated transdermal delivery of β-sitosterol loaded nanostructured lipid nanoparticles for androgenic alopecia. Drug Deliv.

[23] Hebert AA, Friedlander SF, Allen DB (2006). Topical fluticasone propionate lotion does not cause HPA axis suppression. J Pediatr.

[24] Korting HC, Schöllmann C (2012). Topical fluticasone propionate: intervention and maintenance treatment options of atopic dermatitis based on a high therapeutic index. J Eur Acad Dermatol Venereol.

[25] Menter A, Griffiths CE (2007). Current and future management of psoriasis. Lancet.

[26] Aungst BJ, Rogers NJ, Shefter E (1986). Enhancement of naloxone penetration through human skin in vitro using fatty acids, fatty alcohols, surfactants, sulfoxides and amides. Int J Pharm.

[27] Garg J, Pathania K, Sah SP, Pawar SV (2022). Nanostructured lipid carriers: a promising drug carrier for targeting brain tumours. Futur J Pharm Sci.

[28] Murthy A, Ravi PR, Kathuria H, Malekar S (2020). Oral bioavailability enhancement of raloxifene with nanostructured lipid carriers. Nanomaterials (Basel).

[29] El-Tokhy FS, Abdel-Mottaleb MM, Abdel Mageed SS, Mahmoud AM, El-Ghany EA, Geneidi AS (2023). Boosting the in vivo transdermal bioavailability of asenapine maleate using novel lavender oil-based lipid nanocapsules for management of schizophrenia. Pharmaceutics.

[30] Andrade LM, Silva LAD, Krawczyk-Santos AP, de Amorim IC, da Rocha PB, Lima EM (2017). Improved tacrolimus skin permeation by co-encapsulation with clobetasol in lipid nanoparticles: Study of drug effects in lipid matrix by electron paramagnetic resonance. Eur J Pharm Biopharm.

[31] Hussein A, Abdel-Mottaleb MM, El-Assal M, Sammour O (2020). Novel biocompatible essential oil-based lipid nanocapsules with antifungal properties. J Drug Deliv Sci Technol.

[32] Abuelella KE, Abd-Allah H, Soliman SM, Abdel-Mottaleb MM (2023). Skin targeting by chitosan/hyaluronate hybrid nanoparticles for the management of irritant contact dermatitis: In vivo therapeutic efficiency in mouse-ear dermatitis model. Int J Biol Macromol.

[33] Abdel-Mottaleb MM, Neumann D, Lamprecht A (2010). In vitro drug release mechanism from lipid nanocapsules (LNC). Int J Pharm.

[34] El-Tokhy FS, Abdel-Mottaleb MM, El-Ghany EA, Geneidi AS (2021). Design of long acting invasomal nanovesicles for improved transdermal permeation and bioavailability of asenapine maleate for the chronic treatment of schizophrenia. Int J Pharm.

[35] Abdel-Mottaleb MM, Neumann D, Lamprecht A (2011). Lipid nanocapsules for dermal application: a comparative study of lipid-based versus polymer-based nanocarriers. Eur J Pharm Biopharm.

[36] Abdel-Mottaleb MM, Beduneau A, Pellequer Y, Lamprecht A (2015). Stability of fluorescent labels in PLGA polymeric nanoparticles: quantum dots versus organic dyes. Int J Pharm.

[37] Swindell WR, Michaels KA, Sutter AJ, Diaconu D, Fritz Y, Xing X (2017). Imiquimod has strain-dependent effects in mice and does not uniquely model human psoriasis. Genome Med.

[38] Fereig SA, El-Zaafarany GM, Arafa MG, Abdel-Mottaleb MM (2021). Tacrolimus-loaded chitosan nanoparticles for enhanced skin deposition and management of plaque psoriasis. Carbohydr Polym.

[39] Fereig S, El-Zaafarany GM, Arafa MG, Abdel-Mottaleb MM (2022). Boosting the anti-inflammatory effect of self-assembled hybrid lecithin-chitosan nanoparticles via hybridization with gold nanoparticles for the treatment of psoriasis: elemental mapping and in vivo modeling. Drug Deliv.

[40] Abdelbary AA, AbouGhaly MH (2015). Design and optimization of topical methotrexate loaded niosomes for enhanced management of psoriasis: application of Box-Behnken design, in-vitro evaluation and in-vivo skin deposition study. Int J Pharm.

[41] Jain A, Doppalapudi S, Domb AJ, Khan W (2016). Tacrolimus and curcumin co-loaded liposphere gel: synergistic combination towards management of psoriasis. J Control Release.

[42] Xu Q, Liu Z, Cao Z, Shi Y, Yang N, Cao G (2022). Topical astilbin ameliorates imiquimod-induced psoriasis-like skin lesions in SKH-1 mice via suppression dendritic cell-Th17 inflammation axis. J Cell Mol Med.

[43] Abdel-Mottaleb MM, Moulari B, Beduneau A, Pellequer Y, Lamprecht A (2012). Nanoparticles enhance therapeutic outcome in inflamed skin therapy. Eur J Pharm Biopharm.

[44] Abdel-Mottaleb MM, Try C, Pellequer Y, Lamprecht A (2014). Nanomedicine strategies for targeting skin inflammation. Nanomedicine (Lond).

[45] Marchiori ML, Lubini G, Dalla Nora G, Friedrich RB, Fontana MC, Ourique AF (2010). Hydrogel containing dexamethasone-loaded nanocapsules for cutaneous administration: preparation, characterization, and in vitro drug release study. Drug Dev Ind Pharm.

[46] Cavanagh T, Barman SP, Hao T, Leland TB, Thekkedath RV. Preparations of Hydrophobic Therapeutic Agents, Methods of Manufacture and Use Thereof. Google Patents; 2017. Available from: https://patents.google.com/patent/US9822142B2/en. Accessed March 25, 2023.

[47] Joshi M, Patravale V (2008). Nanostructured lipid carrier (NLC) based gel of celecoxib. Int J Pharm.

[48] Elmowafy M, Al-Sanea MM (2021). Nanostructured lipid carriers (NLCs) as drug delivery platform: advances in formulation and delivery strategies. Saudi Pharm J.

[49] Doktorovová S, Araújo J, Garcia ML, Rakovský E, Souto EB (2010). Formulating fluticasone propionate in novel PEG-containing nanostructured lipid carriers (PEG-NLC). Colloids Surf B Biointerfaces.

[50] Rodrigues da Silva GH, Ribeiro LN, Mitsutake H, Guilherme VA, Castro SR, Poppi RJ (2017). Optimised NLC: a nanotechnological approach to improve the anaesthetic effect of bupivacaine. Int J Pharm.

[51] Ahmed S, Govender T, Khan I, Rehman NU, Ali W, Shah SM (2018). Experimental and molecular modeling approach to optimize suitable polymers for fabrication of stable fluticasone nanoparticles with enhanced dissolution and antimicrobial activity. Drug Des Devel Ther.

[52] Ali HR, Edwards HG, Kendrick J, Scowen IJ (2009). Vibrational spectroscopic study of fluticasone propionate. Spectrochim Acta A Mol Biomol Spectrosc.

[53] Sutariya V, Kelly SJ, Weigel RG, Tur J, Halasz K, Sharma NS (2019). Nanoparticle drug delivery characterization for fluticasone propionate and in vitro testing (1). Can J Physiol Pharmacol.

[54] Amasya G, Şengel Türk CT, Badilli U, Tarimci N (2020). Development and statistical optimization of solid lipid nanoparticle formulations of fluticasone propionate. Turk J Pharm Sci.

[55] Doktorovová S, Araújo J, Garcia ML, Rakovský E, Souto EB (2010). Formulating fluticasone propionate in novel PEG-containing nanostructured lipid carriers (PEG-NLC). Colloids Surf B Biointerfaces.

[56] Espinosa-Olivares MA, Delgado-Buenrostro NL, Chirino YI, Trejo-Márquez MA, Pascual-Bustamante S, Ganem-Rondero A (2020). Nanostructured lipid carriers loaded with curcuminoids: physicochemical characterization, in vitro release, ex vivo skin penetration, stability and antioxidant activity. Eur J Pharm Sci.

[57] Pischon H, Radbruch M, Ostrowski A, Volz P, Gerecke C, Unbehauen M (2017). Stratum corneum targeting by dendritic core-multishell-nanocarriers in a mouse model of psoriasis. Nanomedicine.

[58] Roberson ED, Bowcock AM (2010). Psoriasis genetics: breaking the barrier. Trends Genet.

[59] Fang JY, Fang CL, Liu CH, Su YH (2008). Lipid nanoparticles as vehicles for topical psoralen delivery: solid lipid nanoparticles (SLN) versus nanostructured lipid carriers (NLC). Eur J Pharm Biopharm.

[60] Negi P, Sharma I, Hemrajani C, Rathore C, Bisht A, Raza K (2019). Thymoquinone-loaded lipid vesicles: a promising nanomedicine for psoriasis. BMC Complement Altern Med.

[61] Varma SR, Sivaprakasam TO, Arumugam I, Dilip N, Raghuraman M, Pavan KB (2019). In vitro anti-inflammatory and skin protective properties of virgin coconut oil. J Tradit Complement Med.

[62] Silva LA, Andrade LM, de Sá FA, Marreto RN, Lima EM, Gratieri T (2016). Clobetasol-loaded nanostructured lipid carriers for epidermal targeting. J Pharm Pharmacol.

[63] Kim HR, Lee A, Choi EJ, Hong MP, Kie JH, Lim W (2014). Reactive oxygen species prevent imiquimod-induced psoriatic dermatitis through enhancing regulatory T cell function. PLoS One.

[64] Croxford AL, Karbach S, Kurschus FC, Wörtge S, Nikolaev A, Yogev N (2014). IL-6 regulates neutrophil microabscess formation in IL-17A-driven psoriasiform lesions. J Invest Dermatol.

[65] Cooper KD, Hammerberg C, Baadsgaard O, Elder JT, Chan LS, Sauder DN (1990). IL-1 activity is reduced in psoriatic skin Decreased IL-1 alpha and increased nonfunctional IL-1 beta. J Immunol.

[66] Girolomoni G, Mrowietz U, Paul C (2012). Psoriasis: rationale for targeting interleukin-17. Br J Dermatol.

