# Supplementary material for: Fluticasone Propionate-loaded Black Cumin Oil NLCs with Dual Deposition/Permeation Capabilities for Enhanced Psoriasis Management
Source: Adv Pharm Bull. 2026 May 19;16(2):403–15. doi: 10.34172/apb.47080 (PMC13768142; doi:10.34172/apb.47080)
Supplement: Supplementary file 1 [file apb-16-403-s001.pdf]

Adv Pharm Bull 2026;16(2):403-S1
doi:10.34172/apb.47080
https://apb.tbzmed.ac.ir

Supplementary Materials

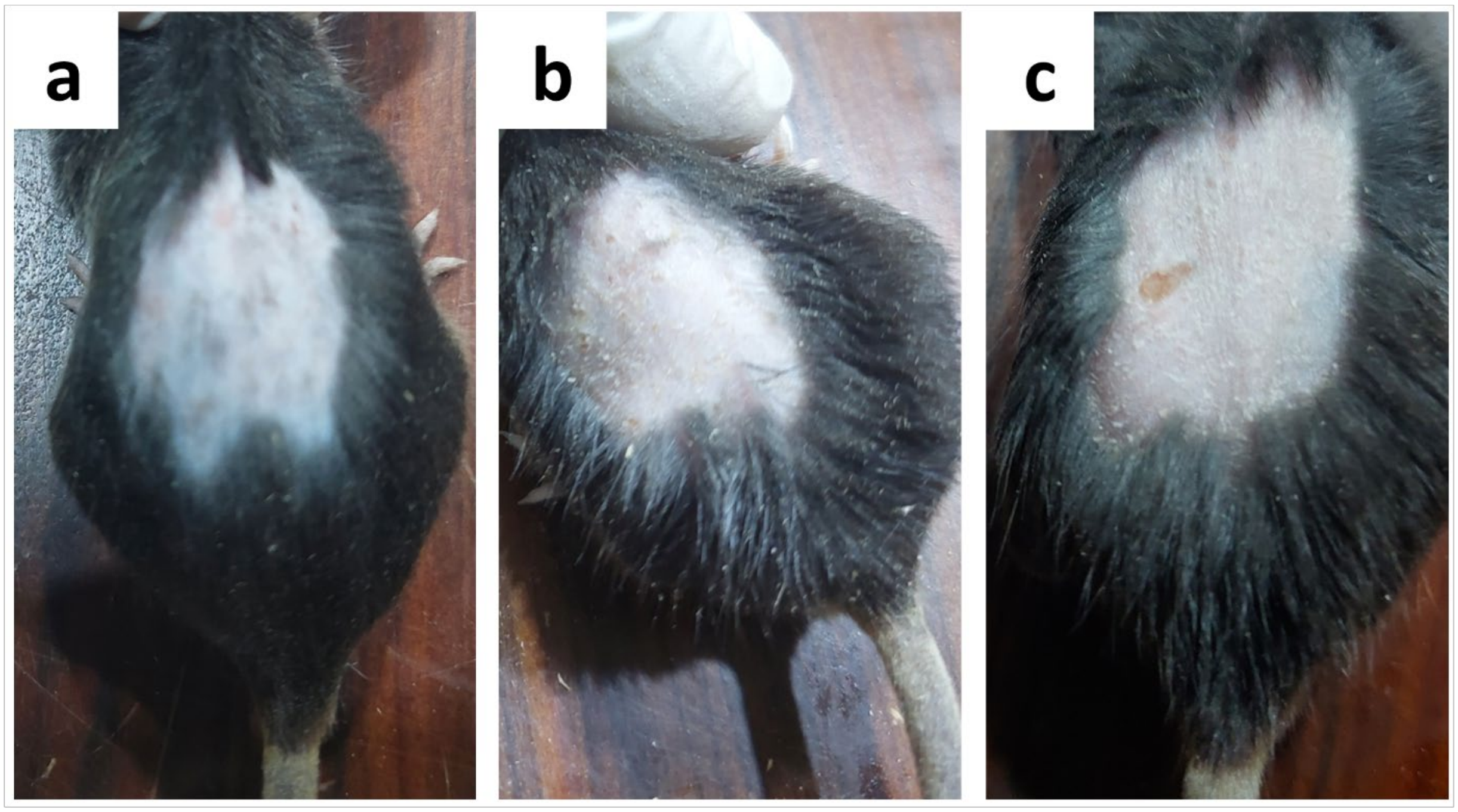

Figure S1. IMQ psoriasis-induced model construction on starting day of the model (a), on day 2 (b), and on day 4 (c) of daily dose application of Aldara® cream (83.5 mg).

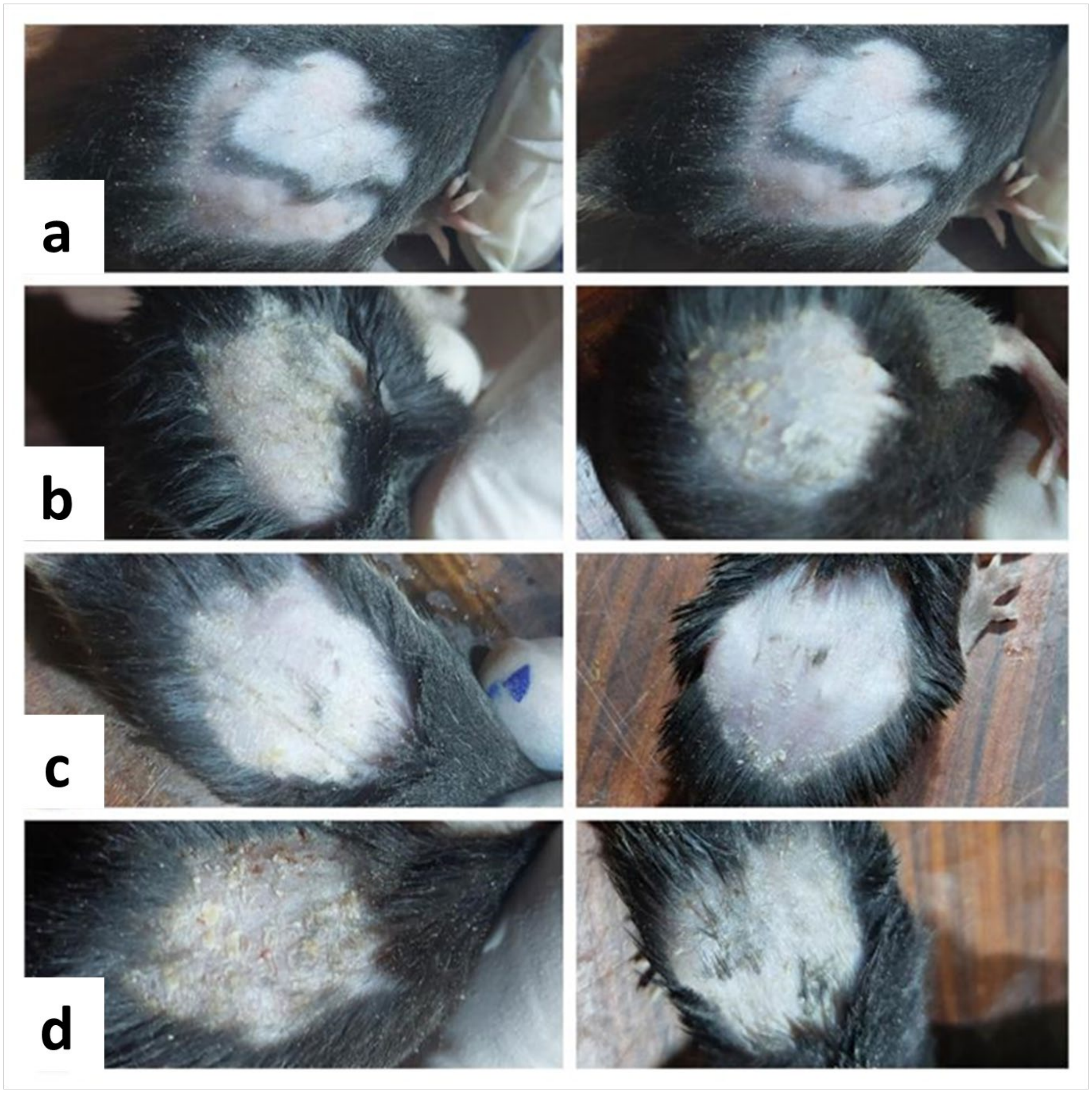

Figure S2. Dorsal mice skin of a representative of each group of the study on day 4 (left) and day 8 (right); a) negative (normal) control b) positive (model) control c) group A; treated with marketed fluticasone cultivate® cream and d) group B; treated with optimized FP-F6 NLC.
